# Recessive antimorph alleles reveal novel functions of the *Opaque1* myosin XI in maize

**DOI:** 10.1093/plphys/kiag605

**Published:** 2026-08-20

**Authors:** Brian Zebosi, Stephanie E Martinez, Kokulapalan Wimalanathan, John Ssengo, Gabriela Brown, Norman B Best, Michelle Facette, Carolyn G Rasmussen, Erik Vollbrecht

**Affiliations:** Department of Genetics, Development and Cell Biology, Iowa State University, Ames, IA 50011, USA; Interdepartmental Genetics and Genomics Graduate Program, Iowa State University, Ames, IA 50011, USA; Botany and Plant Sciences Department, University of California, Riverside, Riverside, CA 92507, USA; Department of Genetics, Development and Cell Biology, Iowa State University, Ames, IA 50011, USA; Interdepartmental Bioinformatics and Computational Biology Graduate Program, Iowa State University, Ames, IA 50011, USA; Interdepartmental Genetics and Genomics Graduate Program, Iowa State University, Ames, IA 50011, USA; Department of Plant Pathology, Iowa State University, Ames, IA 50011, USA; Botany and Plant Sciences Department, University of California, Riverside, Riverside, CA 92507, USA; USDA-ARS, Plant Genetics Research Unit, Columbia, MO 65211, USA; Department of Biology, University of Massachusetts, Amherst, Amherst, MA 01003, USA; Botany and Plant Sciences Department, University of California, Riverside, Riverside, CA 92507, USA; Department of Genetics, Development and Cell Biology, Iowa State University, Ames, IA 50011, USA; Interdepartmental Genetics and Genomics Graduate Program, Iowa State University, Ames, IA 50011, USA; Interdepartmental Bioinformatics and Computational Biology Graduate Program, Iowa State University, Ames, IA 50011, USA

## Abstract

Ideal plant architecture optimizes canopy structure and increases grain yield in maize. However, its underlying genetic mechanisms remain poorly characterized. Two recessive, EMS-induced maize mutants were identified that have reduced stature, opaque kernels, and abnormal subsidiary cell division and are allelic to *Opaque1* (*o1*) which encodes myosin XI-I. These 2 new missense alleles, *o1-2995* and *o1-tan62*, unlike the loss-of-function alleles previously identified, generate O1 protein but paradoxically generate more severe morphological defects. These defects include reduced internode elongation and partial suppression of excessive tassel and ear branching in *ramosa1* (*ra1*)*, ra2*, *and ra3* mutants. We show that *o1-2995* and *o1-tan62* are novel, antimorph alleles of *o1* and play a role in plant growth via internode elongation, subsidiary cell division positioning, leaf patterning, inflorescence development, and overall plant architecture.

## Introduction

Altering maize plant architecture traits has led to significant increases in yield (Tian et al. 2019). Modern hybrids typically have narrow, erect leaves and tassels with few and upright branches that facilitate enhanced photosynthetic efficiency, efficient light interception, and improved tolerance to plant lodging under high planting densities (Pendleton et al. 1968; Lambert and Johnson 1978; Tian et al. 2019). A key aspect of development in grasses is the regulation of branching in grain-producing inflorescences, such as the tassel and ear of maize. Inflorescence branching architecture reflects the presence and differential activity of multiple axillary meristems. Prior research has identified several genes required for initiating axillary meristems and maintaining their determinacy. Some of these genes such as *Barren inflorescence1* (*Bif1*), *Bif2*, *Bif4*, and *Barren stalk 1* (*Ba1*) control axillary meristem initiation and their mutants display decreased tassel branching (McSteen and Hake 2001; Gallavotti et al. 2004; McSteen et al. 2007; Barazesh and McSteen 2008; Galli et al. 2015). In contrast, the *ramosa* genes (*Ra1*, *Ra2*, and *Ra3*) regulate spikelet pair meristem determinacy, and their loss of function mutants produce more tassel and ear branches (Vollbrecht et al. 2005; Bortiri et al. 2006; Satoh-Nagasawa et al. 2006). *Ra1* encodes a C2H2-type zinc finger transcriptional regulator that likely acts as both an activator and repressor of target genes (Vollbrecht et al. 2005; Gallavotti et al. 2010; Eveland et al. 2014). *Ra2* encodes a LATERAL ORGAN BOUNDARIES domain transcription factor (Bortiri et al. 2006). *Ra3* encodes a trehalose-6-phosphate phosphatase whose role in branching is uncoupled from enzymatic activity (Satoh-Nagasawa et al. 2006; Claeys et al. 2019). Using a genetic approach to identify modifiers of *ramosa1* (*ra1*), we uncovered a novel mutant that suppresses tassel branching independent of the *ramosa* pathway and that disrupts a gene encoding a myosin XI-I called *Opaque1* (Wang et al. 2012).

Myosins are molecular motors that transport cargo along cytoskeletal actin filaments (F-actin) and are required for proper cell function. Myosins are important for processes, such as organelle movement, cell shape maintenance, cell polarization, actin organization, and signal transduction (Madison and Nebenführ 2013). The myosin protein structure typically consists of 3 domains: the head, neck, and tail (Foth et al. 2006; Tominaga and Nakano 2012). The head motor domain functions in actin binding and in ATP binding and hydrolysis for motor activity, the neck domain contains a linker that influences motor step size, while the tail binds cargo and facilitates dimerization (Nebenführ and Dixit 2018). Myosins are highly conserved across many eukaryotic species, including mammals, fungi, and plants (Richards and Cavalier-Smith 2005). Two distinct myosin groups (VIII and XI) are specific to plants and present as several subfamilies due to repeated rounds of whole-genome duplications (Foth et al. 2006; Mühlhausen and Kollmar 2013; Nebenführ and Dixit 2018). Genetic studies and functional characterization of mutants of these plant-specific myosins indicate that they are involved in many processes, including organelle translocation, cytoplasmic streaming, cytokinesis, tip growth, exocytosis, and endocytosis (Golomb et al. 2008; Peremyslov et al. 2010; Tominaga and Nakano 2012; Ueda et al. 2015; Nebenführ and Dixit 2018; Zhang et al. 2019; Olatunji et al. 2023; Chocano-Coralla and Vidali 2024).

Arabidopsis myosin XI-I isoforms (myosin XI-Is) are localized to the nuclear envelope and act as linkers between the nucleus and actin cytoskeleton through a linker of nucleoskeleton and cytoskeleton complex (Tamura et al. 2013). Arabidopsis myosin XI-I mutants (*Atxi-1*/*kaku1*) have aberrant nuclear envelopes. Mutants also have impaired nuclear shape and movement, thus emphasizing the vital role of myosins in nuclear positioning (Tamura et al. 2013; Muroyama et al. 2020).

Myosin XI-I in maize is encoded by *Opaque1* (or *Opaque endosperm1*, *O1*) (Wang et al. 2012). The classical *o1* mutant generates an opaque endosperm in contrast to the translucent endosperm in nonmutant kernels (Emerson et al. 1935; Neuffer et al. 1968). While many other *opaque* mutants are characterized by reduced accumulation of zeins, the primary storage proteins in the endosperm (Gibbon and Larkins 2005; Larkins et al. 2017; Zhang et al. 2018), *o1* does not accumulate zeins aberrantly (Nelson et al. 1965; Hunter et al. 2002). Genetic studies in maize indicate that O1-myosin XI-I is required for protein body formation during seed development. Further, heterologous expression of a dominant-negative *O1-tail* transgene in tobacco slowed endoplasmic reticulum (ER) movement (Wang et al. 2012). Finally, *opaque1* mutants have phragmoplast guidance defects leading to asymmetric cell division defects. Unlike *kaku1* mutants, however, no nuclear movement abnormalities were observed during or prior to abnormal, *o1* asymmetric divisions (Nan et al. 2023). None of these previous studies reported growth or shoot development defects in *o1* mutants.

Here, we functionally characterized 2 new alleles of *Opaque1*, a myosin XI-I (Zm00001eb193160). One mutant allele, ramosa1 suppressor locus*12.2995 (rsl*-12.2995), was identified from a *ramosa1* EMS enhancer–suppressor screen as a mutant with reduced stature, abnormal subsidiary cell division, opaque kernels, and impaired tassel, ear, and vegetative shoot development. The rsl*-12.2995 mutation was mapped to the *O1* locus using map-based cloning and whole-genome sequencing (WGS) and subsequently named *o1-2995*. The other mutant allele, *tangled62* (*o1-tan62*), was identified from a forward genetics EMS screen for plants with abnormal subsidiary cell divisions. Allelism tests confirmed both as new *o1* alleles that in genetic analyses behaved as recessive antimorphs. Thus, *o1-2995* and *o1-tan62* are novel alleles of *Opaque1* (*O1*) that regulate plant growth, architecture, and asymmetric division positioning in maize.

## Results

### The *o1-2955* mutant shows aberrant shoot and inflorescence architecture

**Figure 1:**
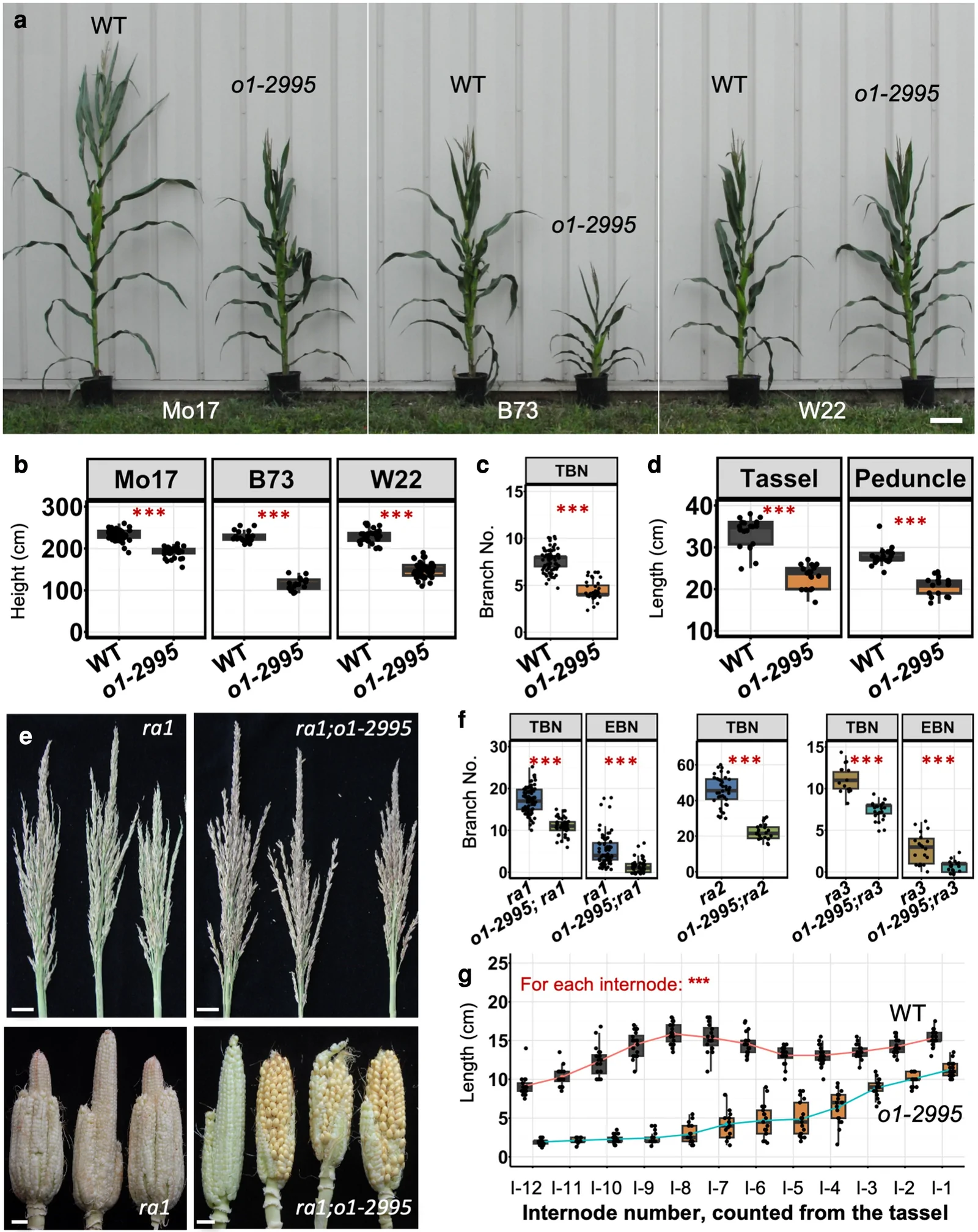
Phenotypic characterization of *o1-2995* and of its interaction with *ramosa* (*ra*) pathway genes. a and b) Plant height across inbred line (B73, Mo17, and W22) introgressions for normal siblings (WT, *n* = 47, 20, 21 for Mo17, B73, W22, respectively) and *o1-2995* mutants (*n* = 39, 17, 39 for Mo17, B73, W22, respectively). Bar, 20 cm. c) TBN of normal siblings (WT, *n* = 75) and *o1-2995* single mutants (*n* = 33). d) Peduncle length and whole tassel length of normal siblings (WT, *n* = 30) and *o1-2995* mutants (*n* = 30) in B73. e and f) *o1-2995* and its interaction with *ra1*, *ra2*, and *ra3* in B73. e) Representative tassels and ears of the single and double mutants reported in (f). Bars, 2 cm. f) Left, TBN and EBN of *ra1* single mutants and *ra1;o1-2995* double mutants. Center, TBN of *ra2* single mutants and *ra2;o1-2995* double mutants. Right, TBN and EBN of *ra3* single mutants and *ra3;o1-2995* double mutants. g) Length of several internodes of the main shoot in normal siblings (WT, left, successive means connected by red line, *n* = 30) and *o1-2995* mutants (right, successive means connected by blue line, *n* = 30) in B73. I-1 denotes the internode nearest the tassel, and I-12 is 11 internodes away, near the soil line. Asterisks (***), significant difference at *P* < 0.001.

Genetic and genomics studies indicate that 3 *Ramosa* genes in maize (*Ra1*, *Ra2*, and *Ra3*) regulate branching in grass inflorescences by modulating shared and discrete developmental modules (Vollbrecht et al. 2005; Bortiri et al. 2006; Satoh-Nagasawa et al. 2006; Eveland et al. 2014). To identify *ramosa1* (*ra1*) mutant modifiers, we performed an EMS mutagenesis screen of *ra1-63* mutants (Vollbrecht et al. 2005) in the Mo17 genetic background. In the M2 generation, we identified a short-statured mutant that suppressed *ra1*-induced tassel and ear branching, named it ramosa suppressor locus*-12.2995 (rsl*-12.2995) and later renamed it *o1-2995*. In F2 and backcross populations, *o1-2995* segregated as a single recessive locus (wild type [WT]: *o1-2995*, 48:22 [χ^2^, *P* = 0.21] and 22:18 [χ^2^, *P* = 0.53], respectively). *o1-2995* single mutants also showed reduced stature after backcrossing 6 times into B73 and 2 times into W22 with coefficient of variation for plant height measurements ranging from 5% to 13% (Fig. 1a and b; Table S1). The *o1-2995* mutant's effect on plant height was completely penetrant in all 3 genetic backgrounds, but its expressivity varied. Compared to their normal siblings, field-grown *o1-2995* mutants were shortest in B73, suggesting that especially in the B73 genetic background, genetic modifiers interact with *O1* in its effect on plant height. All subsequent phenotyping was carried out using the well-introgressed B73 stocks.

In B73, tassel branch number (TBN) was reduced in the *o1-2995* single mutant, which produced 51.1% fewer tassel branches than its WT siblings (Fig. 1c). Length of the tassel peduncle and tassel length exhibited a 70% and 78.1% reduction, respectively, in mutants compared to WT siblings (Fig. 1d).

Because *o1-2995* was isolated from a *ra1-63* mutant suppressor genetic screen, we constructed double mutants between *o1-2995* and all 3 *ramosa* pathway mutants (*ra1*, *ra2*, and *ra3*) and also produced 1 triple mutant (*ra1*;*ra2*;*o1-2995*). We compared the higher order mutant phenotypes to the respective single mutants to query whether *O1* and *Ra* genes function in an additive, synergistic, or epistatic manner with one another. In *ra1*, *ra2*, or *ra3* mutants, additional long branches form in both the ear and in the tassel. As stated earlier, the *o1-2995* single mutant reduced TBN by 51.1% as compared to its WT siblings (Fig. 1c). *o1-2995* single mutants did not reduce ear branch number (EBN) as the normal ear is unbranched. In double mutant populations with *ra1-63*, the *o1-2995* mutant reduced TBN by 36.5% and EBN by 72.3% (*P* < 0.001, Student's *t*-test), as compared to *ra1-63* single mutants (Fig. 1e and f, left). Thus, in combination with *ra1-63*, the *o1-2995* mutant slightly reduced overall branching. The *ra2-R;o1-2995* double mutant combination was similar with a 51.4% reduction in TBN in the double mutant as compared to the *ra2-R* single mutant (Fig. 1f, center). In mutant combinations with *ra3*, *o1-2995* also suppressed tassel and ear branching; the *ra3-R*;*o1-2995* double mutants exhibited 33.4% and 76.6% reductions in TBN and EBN, respectively, compared to *ra3-R* single mutants (Fig. 1f, right). Thus, for 2 double mutants (*ra1-63*;*o1-2995* and *ra3-R*;*o1-2995*), the inflorescence branching phenotypes suggested a nonadditive genetic interaction wherein the effect of *o1-2995* was weaker in *ra1* or *ra3* mutant background than in WT. The *ra2-R*;*o1-2995* phenotype, on the other hand, suggested additivity and lacked clear evidence of epistasis, as if *o1-2995* affected growth more generally without specifically affecting aspects of meristem determinacy regulated by *ramosa2*. As a final test, we examined *ra1;ra2* double mutants which interact synergistically by more completely reducing activity of the *ramosa* pathway, leading to profusely branched ears (Vollbrecht et al. 2005). The (*ra1-63;ra2-R;o1-2995*) triple mutants had substantially less ear branching and smooth patches on the ear axis, suggesting a synergistic interaction with *o1-2995*, because the bare patches are a novel phenotype not seen in any of the 3 single or double mutants (Fig. S1). These multimutant studies imply that *O1* and *ramosa* genes act in either independent or converging genetic pathways and are unlikely to participate together in a simple linear pathway to regulate inflorescence branching.

### Map-based cloning and identification of *o1-2995* as a novel allele of *Opaque1*

**Figure 2:**
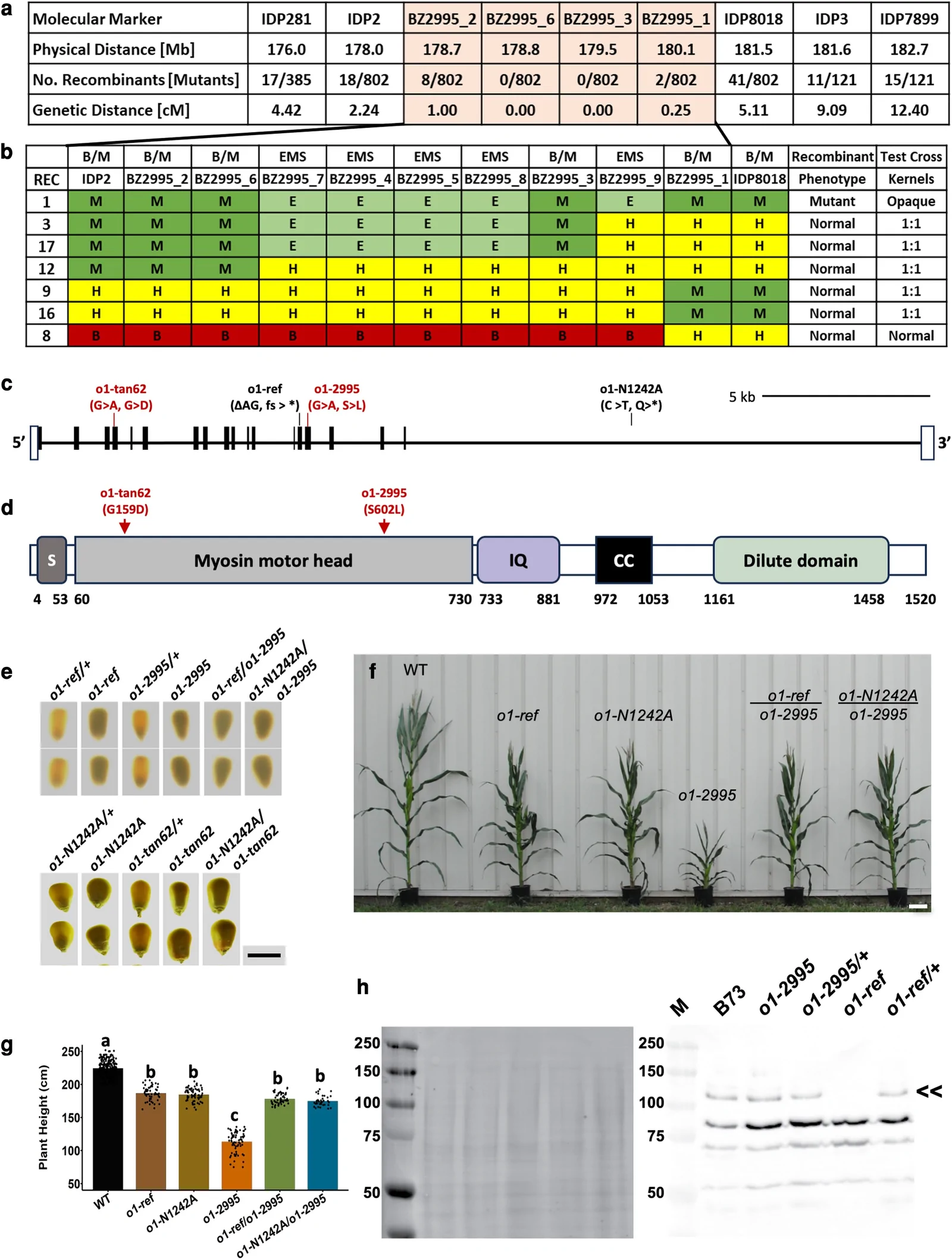
Map-based cloning, *o1-2995* and *o1-tan62* complementation tests, and antimorph allele relationships. a and b) Map-based cloning of *o1-2995*. (a) Highlighted interval (shaded) is ∼1.5 Mb with 28 genes and no B73/Mo17 polymorphism. (b) WGS-aided recombination mapping. E, EMS-like SNP; M, homozygous Mo17; H, heterozygous Mo17/B73; B, homozygous B73. The new interval contained 8 genes and only 1 exonic, EMS-like SNP (G > A), in the *o1* gene. c) *o1* intron–exon gene structure (from NCBI) showing relative mutation sites of *o1-2995* and *o1-tan62* and of null alleles *o1-N1242A* and *o1-ref* (Wang et al. 2012) used in this study. d) Maize O1 protein domain structure and relative locations of *o1-2995* and *o1-tan62* missense mutations in the myosin head domain. S, SH3-like domain; IQ, region of 6 IQ domains; CC, coiled-coil domain. e to g) Complementation test for plant height and kernel phenotypes. e) Kernels of indicated genotypes, illuminated from below on a light box; *o1-2995* and *o1-tan62* fail to complement endosperm phenotype of null alleles *o1-ref* and *o1-N1242A*. Bar, 1 cm. f and g) Field-grown plants of indicated genotypes in B73; *o1-2995* fails to complement plant height phenotype of null alleles *o1-ref* and *o1-N1242A*. Bar, 20 cm. h) Ponceau stained membrane (left) and anti-O1 western blot (right) of membrane and membrane-associated proteins isolated from the stomatal division zone of WT (B73) and *o1* mutant plants using anti-O1 antibodies (Nan et al. 2023). Lower bands are nonspecific. M, marker lane (kD). Double arrowhead, migration position of the O1 protein.

To identify the causative mutation responsible for the rsl*-12.2995 (*o1-2995*) mutant phenotype, we used map-based cloning methods. Bulked segregant analysis using genotyping-by-sequencing (BSA-GBS) mapped the *o1-2995* mutation to the long arm of chromosome 4 (Fig. S2), within a peak containing 989 genes. Mapping using publicly available molecular markers identified a 1.5 Mb interval containing 28 genes (Fig. 2a) which, in nonrepetitive regions, contained no DNA sequence polymorphisms between B73 and Mo17. We therefore performed WGS of a single mutant plant and identified EMS-induced single nucleotide polymorphisms (SNPs) in the interval. Fine-mapping in a second, larger (2,289 plants) population indicated that the causal mutation was within a physical distance of ∼0.6 Mb that contained only one gene, the previously cloned *Opaque1* (*O1*) (Fig. 2b). We therefore phenotyped kernels from our segregating populations and found an opaque kernel phenotype cosegregated with the rsl*-12.2995 mutant plant phenotypes.

**Figure 3:**
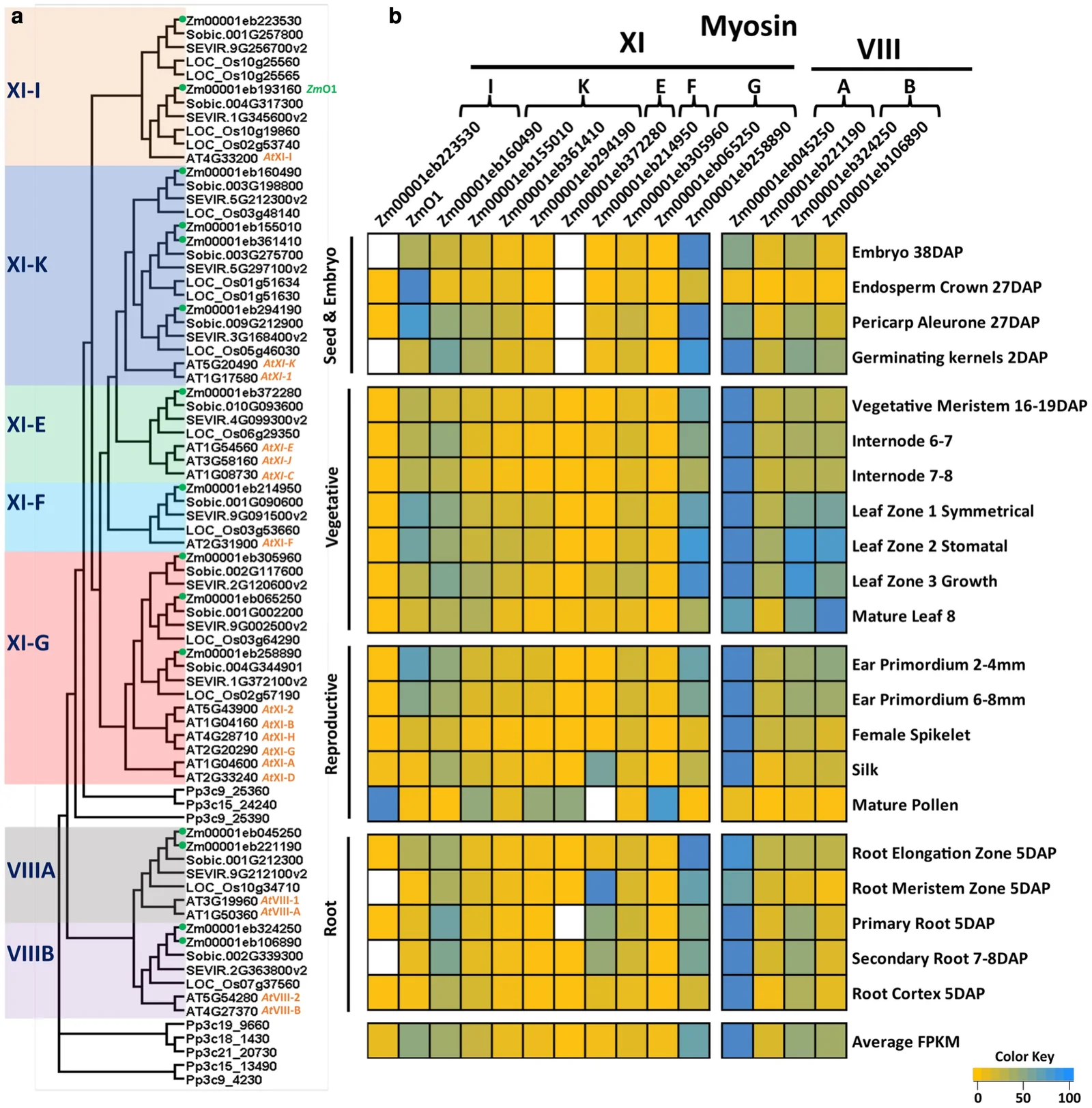
Myosin XI and VIII genes and their expression in various plant species. a) Phylogenetic analysis of all plant myosins XI and VIII across several species. Zm, maize; Sobic., *Sorghum bicolor*; SEVIR, *Seteria viridis*; Os, rice; AT, Arabidopsis; Pp, *Physcomitrella patens* (moss) as an out-group. b) Relative transcript expression levels of myosin XI and VIII genes in different maize tissues; from Walley et al. (2016).

Sanger sequencing of the *O1* locus in *o1-2995* confirmed the single nucleotide change (G > A) in the 15th exon (Fig. 2c), causing a missense mutation (S602L) within the myosin head domain of O1 (Fig. 2d). S602 is a nearly invariant residue near the actin-binding motif: across the 72 myosin VIII and myosin XI genes from flowering and nonflowering plants, summarized in Fig. 3, 71 genes encode an S in this position and 1 encodes a T.

**Figure 4:**
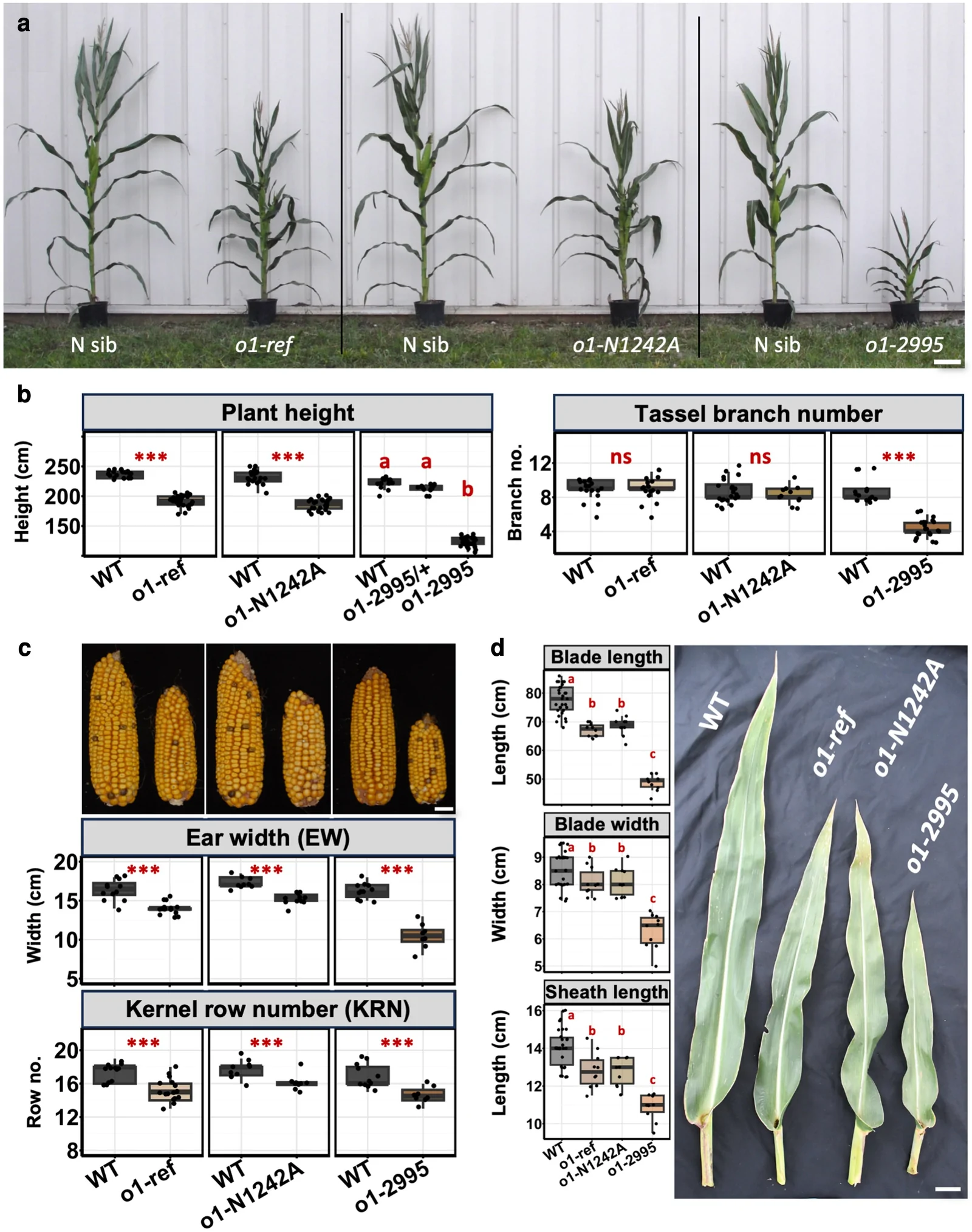
Shoot phenotypes of *o1* mutant alleles in B73. a) Plant height of indicated genotypes and WT siblings. Bar, 20 cm. b) Plant height (left) and TBN (right) of indicated genotypes and WT siblings. c) Ears (top) and quantification of ear size traits of indicated genotypes and WT siblings. Bar, 2 cm. d) Quantification of leaf traits (left) and images of leaves (right) of WT (B73) and indicated mutant genotypes. Bar, 5 cm. Error bars, standard error; asterisks, significance at *P* < 0.001 by *t*-test; letters a, b, c, significance by ANOVA; ns, no significant difference.

To confirm the genetically linked, EMS-induced mutation in *O1* as causal of the *o1-2995* phenotype, we performed complementation tests with previously identified null mutants, *o1-ref* and *o1-N1242A* (Wang et al. 2012). *o1-2995* failed to complement the *o1* null alleles for kernel opaqueness (Fig. 2e). Interestingly, we also discovered that each null mutant, *o1-ref* and *o1-N1242A*, showed slightly reduced plant height (Fig. 2f and g), a previously unreported phenotype. Moreover, the reduced plant height of the compound heterozygous mutants (*o1-ref*/*o1-2995* and *o1-N1242A*/*o1-2995*) was similar to *o1-ref* or *o1-N1242A* homozygotes. Plant height was greatly reduced for *o1-2995* mutants (Fig. 2f and g; see also Fig. 4a). Thus, the *o1-2995* allele generates a stronger mutant phenotype when homozygous than do null mutant alleles *o1-ref* or *o1-N1242A*. These data imply that *o1-2995* functions as a formally recessive but dose-dependent, antimorphic, allele of *O1.*

To investigate if the nonsynonymous point mutation in *o1-2995* altered O1 protein levels, we isolated proteins from the stomatal division zone of the leaf and used an O1-specific antibody to compare amounts of protein produced between *o1-ref* and *o1-2995*. As expected, a band was detected in the normal inbred B73 plants and WT, heterozygous siblings of *o1-ref* and *o1-2995*, while no band corresponding to the size of the O1 protein was detected in *o1-ref* mutant plants (Fig. 2h). In contrast, in *o1-2995* mutants, a band of the expected size was detected, indicating the mutant produces a protein (Fig. 2h). These data indicate that *o1-2995*, which expresses myosin XI-I containing a S602L mutation in the motor domain, confers a stronger mutant phenotype than *o1* null mutants, which express no or very little myosin XI-I protein.

### *o1-tangled62* is another novel allele of *Opaque1*

An additional mutant allele of *o1* was identified through a forward genetics screen to identify mutants with division plane orientation defects. A recessive mutant with aberrant subsidiary cells was initially identified as *tangled62* (*tan62*) (Fig. S3). Due to the similarity of the subsidiary cell division defect compared to *o1* mutants as well as an opaque kernel phenotype (Wang et al. 2012; Nan et al. 2023), complementation tests were performed with *o1-N1242A* mutants. Across 11 independent crosses, *tan62* failed to complement the *o1-N1242A* opaque kernel phenotype, suggesting that *tan62* is allelic to *o1* (Fig. 2e). Sequencing of the *tan62* cDNA and DNA extracted from 3 additional *tan62* mutants revealed a single nucleotide change (G > A) in the fourth exon (Fig. 2c), causing a missense mutation (G159D) within the myosin motor head domain (Fig. 2d). G159 is an invariant residue in the head domain of all myosins VIII and XI across flowering and nonflowering plants surveyed in Fig. 3, located in the ATP binding pocket. This type of mutation is predicted to be deleterious but still produce protein, similar to O1-2995. Therefore, *tan62* was renamed to *o1-tan62*.

### Genome-wide identification and functional analysis of myosin XIs in maize

To consider novel *O1* alleles in the context of the genome-wide diversification of myosins in maize and other plants, we constructed a phylogenetic tree and annotated it with expression data using publicly available sources (Wang et al. 2012; Walley et al. 2016). Using BLAST searches based on Arabidopsis myosin XI and VIII protein sequences, we included all relevant amino acid sequences from Arabidopsis, rice, sorghum, *Setaria*, and as an out-group, *Physcomitrium patens* (moss) (Fig. 3a). Within the myosin XI clade, Arabidopsis, maize, and rice have 13, 12, and 11 genes, respectively, while Setaria and sorghum have 10 myosin XIs. In general (Fig. 3a), the myosin XIs are further clustered into 5 distinct subclades (XI-I, XI-K, XI-E, XI-F, and XI-G) based on the Arabidopsis nomenclature (Reddy and Day 2001; Avisar et al. 2008; Peremyslov et al. 2011). *Opaque1* (*O1*) belongs to the myosin XI-I clade, which comprises 2 genes in maize, Setaria, and sorghum, contrasting to 1 in Arabidopsis and 4 in rice. Spatial expression differences among the different maize myosins (Fig. 3b) were annotated from publicly available expression data (Wang et al. 2012; Walley et al. 2016). The 2 myosin XI-I genes in maize (Zm00001eb223530 and *O1*) exhibited opposite expression patterns. Notably, *O1* is expressed more broadly and more highly than Zm00001eb223530 in most tissues surveyed except mature pollen, which implies functional diversification between the 2 genes and suggests they function nonredundantly.

### Effects of different *o1* mutant alleles on plant shoot phenotypes and in different environmental conditions

*Opaque1* has been studied for over 50 years as a classical endosperm mutant (Neuffer et al. 1968; Wang et al. 2012), yet plant shoot phenotypes in *o1* loss-of-function mutants, pointing to a role in asymmetric cell divisions in the leaf, have only recently been reported (Nan et al. 2023). Because *o1-2995* antimorph allele mutants had altered shoot development phenotypes, such as tassel branching and plant height (Fig. 1) and both *o1-2995* and *o1-tan62* failed to complement the endosperm phenotype of *o1* null alleles (Fig. 2f), we hypothesized that additional shoot phenotypes would be affected by various alleles and tested this hypothesis using our mutant stocks in the B73 genetic background.

### Field-grown plant phenotypes indicate a role for *O1* in plant height and ear growth

In side-by-side field growth experiments comparing single mutants of the antimorphic *o1-2995* allele and the null alleles *o1-ref* and *o1-N1242A*, the *o1-2995* plants were again dramatically smaller (45.8% height reduction) than *o1-2995/+* or homozygous +/+ siblings. The *o1-ref* and *o1-N1242A* mutants also showed height reduction compared to their WT siblings, although the reduction was less severe than in *o1-2995* (18.7% and 19.6% reduction, respectively) (Fig. 4a and b). On the other hand, *o1-2995* mutants had 3 to 5 fewer tassel branches, but *o1-ref* and *o1-N1242A* null mutant alleles had no significant effects on TBN relative to their WT siblings (Fig. 4b, right). Finally, the length and width of ears, and kernel row number, were significantly reduced in all mutants compared to their WT siblings, with more abnormal phenotypes observed in *o1-2995* mutants than in the null allele mutants (Fig. 4c). These data suggest that *O1* plays a greater role in plant height and ear growth than in tassel branching.

### Greenhouse-grown plant phenotypes suggest *O1* is responsive to environmental conditions

Greenhouse growth experiments also showed reductions in plant height in some of *o1* mutants compared to WT siblings but with more variability. When *o1-2995* mutants were grown in the greenhouse during the shorter days of winter 2018, mutants were significantly shorter than their WT siblings (Fig. S4a), although proportionally less so (20.2%) compared to the reduction in field-grown plants (45.8%). In a separate greenhouse experiment performed in 2024 where total plant height again showed greater variance, both *o1-2995* and *o1-tan62* mutants had a significant reduction in plant height of 37.8% and 17.1%, respectively, compared to WT siblings, while *o1-N1242A* null mutants did not, when measured at the whole plant level (Fig. S4b). Similarly, peduncle length, tassel length, and TBN were unaffected in *o1-N1242A* but were generally reduced in *o1-tan62* and *o1-2995* mutants. Thus, *o1-2995* mutants grown in greenhouse conditions, as compared to field-grown mutants, showed more reduced stature in one environment (2018) than in another (2024), and the stature of *o1-N1242A* mutants was relatively unaffected in the 2024 greenhouse environment, unlike in the field. These data suggest environmental conditions may modulate the severity of phenotypic effects, but under all conditions tested *o1-2995* and *o1-tan62* had more reduced stature compared to both WT siblings and to null mutants *o1-ref* and *o1-N1242A*.

### Plant height reduction is due to compressed internodes

To understand the basis of reduced stature of the *o1-2995* mutants, we counted the number of leaves formed and measured the size of internodes. Our results showed that the normal siblings and mutants generated similar numbers of leaves (normal, *n* = 100, 20.2 leaves; *o1-2995*, *n* = 42, 19.1 leaves; *P* = 0.072) but that in mutants, internode lengths (Figs. 1g and S4c) and diameters (Fig. S5) were compressed, with a 26% to 83% internode length reduction (*P* < 0.0001) compared to WT siblings depending on the observed internode. The reduction in internode length varied but was more pronounced in the lower internodes (with 78.9% and 83.2% reduction) than in the internodes above the ear and toward the tassel. Similarly reduced internode lengths were also observed in *o1-tan62* mutants (Fig. S4b). Thus, the plant height reduction in the *o1-2995* and *o1-tan62* mutants is due to impaired internode elongation.

### *O1* functions in leaf cell expansion and leaf development

In side-by-side field growth experiments comparing single mutants of the antimorphic *o1-2995* allele and the null alleles *o1-ref* and *o1-N1242A*, we observed generally smaller and narrower leaves in all mutants as compared to their normal siblings. Mirroring the trend observed for plant height, length of leaf blades and sheaths and width of leaf blades were slightly reduced in the null mutants, and more so in *o1-2995* antimorph mutants (Figs. 4d and S6a). To determine the basis of small leaf size, we examined *o1-2995* mutants by making abaxial leaf epidermal impressions from leaf 16 using super-glue and observed the impressions under a compound microscope (Fig. S6b). Compared to their WT siblings, *o1-2995* mutants had significantly more cells per microscope field of view due to reduced cell size (Fig. S6c and d). Thus, the *o1-2995* mutation impairs cell expansion and growth.

In addition, *o1-2995* mutants displayed aberrant leaf patterning. Especially when grown in the field, *o1-2995* mutants had ectopic tissue outgrowths extending from the ligule into the leaf midrib (Fig. S7a to c). To further investigate if *o1-2995* mutation also affected vein development, we examined acidified phloroglucinol stained cross sections of distal midribs of adult leaf 10 under a dissecting microscope. Mutants had a markedly reduced volume of clear cells, an adaxial tissue comprising the bulk of the midrib, resulting in correspondingly reduced midribs and smaller veins compared to their WT siblings (Fig. S7d to g). The ectopic outgrowths were not observed in *o1-2995* or *o1-tan62* mutants grown in the greenhouse, or in any *o1* null mutants grown under any conditions. These data indicate that the antimorph *o1-2995* allele disrupts ligule and midrib patterning and development and does so more severely under field grown conditions.

### *O1* antimorph alleles show severe subsidiary cell division defects

**Figure 5:**
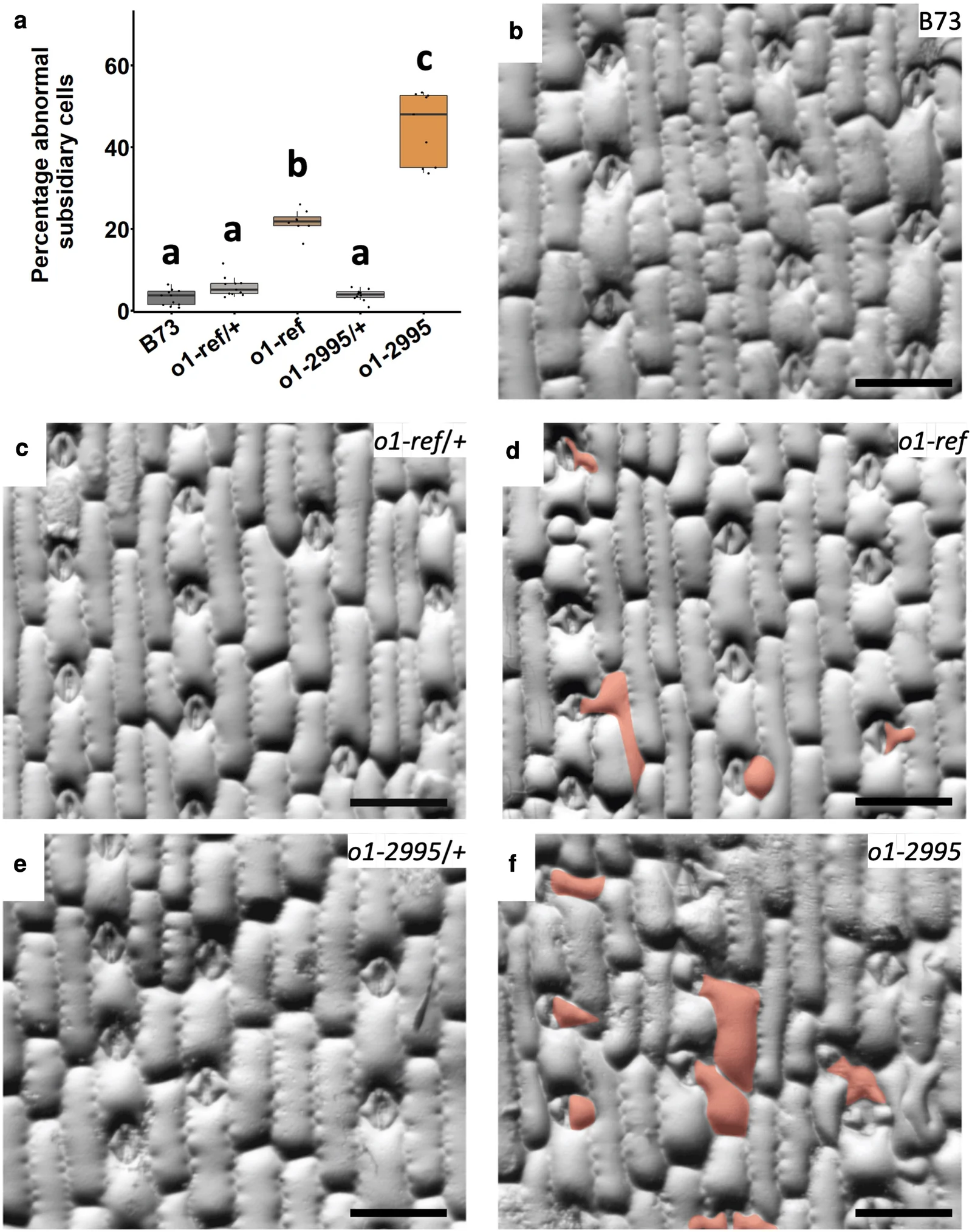
*O1* mutants exhibit abnormal subsidiary cell division. a) Abnormal subsidiary cells were counted in B73 WT, *o1-2995*, *o1-ref*, and their corresponding WT siblings. Genotypes that are not significantly different at *P* = 0.05 via 2-sided *t*-test are noted by the same letter. Different letters a, b, c indicate significantly different. Methacrylate impressions on leaf 4 of (b) B73 (c) *o1-ref/+* and (d) *o1-ref*, (e) *o1-2995/+*, and (f) *o1-2995*. Abnormal cells are highlighted in brown. Bar, 100 *µ*m.

*o1* null mutant alleles (*o1-ref* and *o1-N1243*) were reported to cause kernel opaqueness, impaired ER motility, irregular protein body shape, and aberrant stomatal subsidiary cell division (Wang et al. 2012; Nan et al. 2023). We showed previously that *o1* null mutants have abnormal subsidiary cells and defective phragmoplast guidance (Nan et al. 2023). From epidermal glue impressions of leaf 4, the *o1-ref* allele had 20% abnormal subsidiary cells, similar to previously published results (Nan et al. 2023), while plants homozygous for the antimorph *o1-2995* allele had ∼45% abnormal subsidiary cells (Fig. 5a to f). *o1-N1242A* mutants had ∼20% abnormal subsidiary cells (*n* = 11 plants), also similar to previous reports (Nan et al. 2023), while *o1-tan62* mutants had ∼30% abnormal subsidiary cells (*n* = 15 plants) in juvenile leaf 2 (Fig. S3a). Thus, both antimorph alleles *o1-2995* and *o1-tan62* more severely disrupt cell divisions for subsidiary cells than *o1* null mutants.

**Figure 6:**
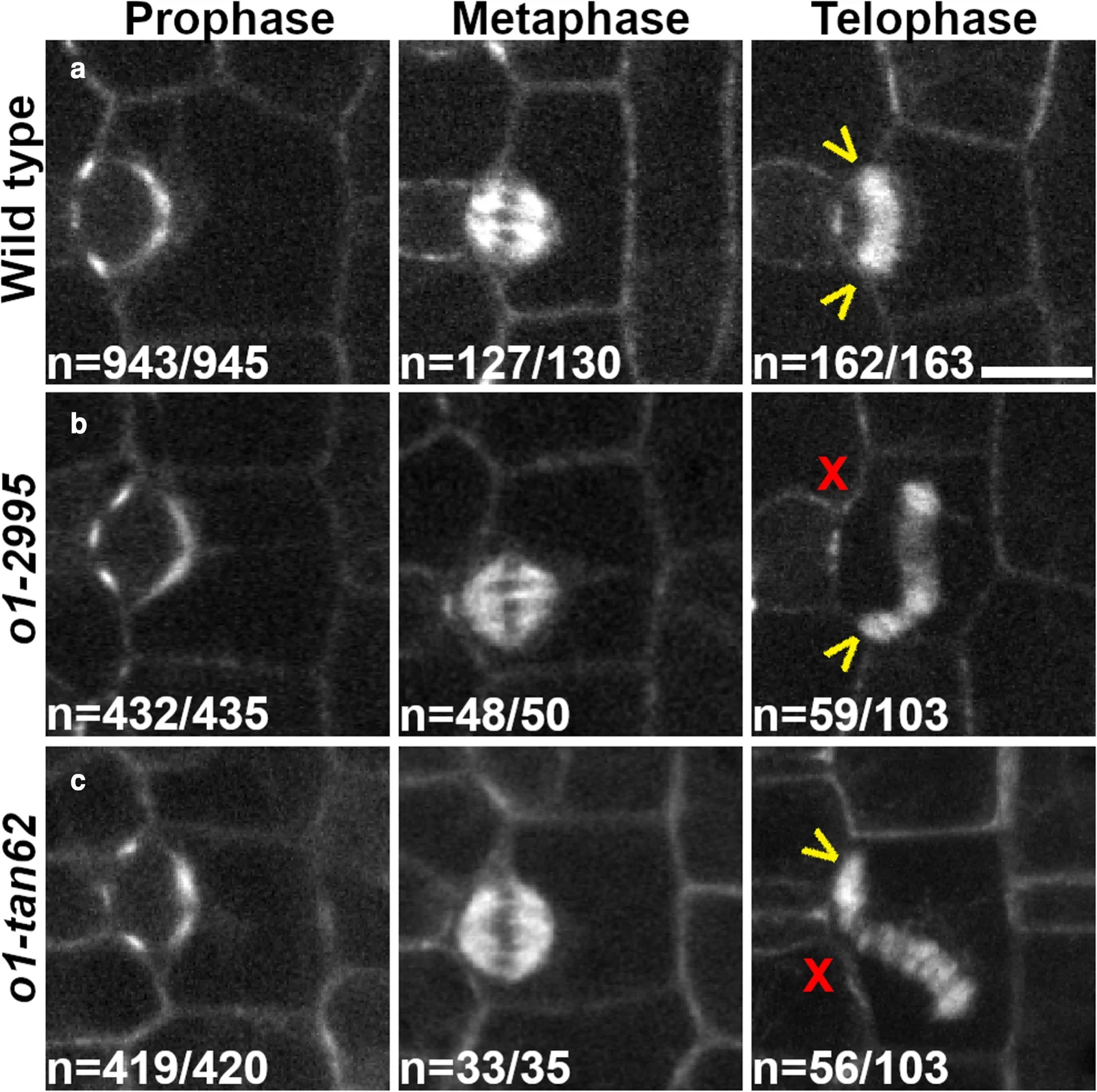
*o1-2995* and *o1-tan62* have phragmoplast guidance defects. Representative micrographs of each cell cycle stage in plants expressing a TUBULIN fluorescent marker. Three plants of b.) *o1-2995*, c.) *o1-tan62*, and a.) their respective WT siblings were analyzed. For prophase, counts indicate the total number of cells with normal preprophase bands. WT sibling of *o1-2995*: *n* = 442/442 cells; WT sibling of *o1-tan62*: *n* = 501/503 cells. For metaphase, counts indicate the total number of cells with normal spindle morphology. WT sibling of *o1-2995*: *n* = 80/83 cells; WT sibling of *o1-tan62*: *n* = 47/47 cells. For telophase, count in WT indicates the total number of oriented phragmoplasts. WT sibling of *o1-2995*: *n* = 90/91 cells; WT sibling of *o1-tan62*: *n* = 72/72 cells. Telophase cell count in *o1-2995* and *o1-tan62* indicates total number of cells with misoriented phragmoplasts. Arrows = oriented phragmoplast. X letters = disoriented phragmoplast. Bar, 10 *µ*m, applies to every image.

To determine when the asymmetric division of the subsidiary mother cell becomes misoriented, *o1-2995* and *o1-tan62* were crossed to a live cell marker for microtubules (YFP-TUBULIN or CFP-TUBULIN [Mohanty et al. 2009]). Then, 3 WT siblings and 3 mutants were dissected to reveal the asymmetric division zone and imaged. In WT siblings, premitotic microtubule structures were oriented correctly (Fig. 6a). Similarly, spindles and preprophase bands in *o1-2995* and *o1-tan62* were similar to WT (Fig. 6b and c). In *o1-2995* and *o1-tan62* mutants, phragmoplasts were often misoriented (∼60% for both). These data indicate that *o1-2995* and *o1-tan62* exhibit phragmoplast guidance defects that are similar to, yet more frequent and severe than, those of previously described *o1* null mutants (Nan et al. 2023).

## Discussion

Similar to *o1* mutants, triple and quadruple mutants in class XI myosins in Arabidopsis generate smaller plants, with reduced cell expansion, short roots, aberrant organelle movement, and division plane positioning defects (Peremyslov et al. 2010; Madison et al. 2015; Abu-Abied et al. 2018; Huang et al. 2024). Often, myosin XI mutants have shortened root hairs or pollen tubes indicating cell elongation defects (Madison et al. 2015; Peremyslov et al. 2015). In *P. patens*, RNAi directed at the only 2 myosin XIs generated very small plants with round cells also indicating a critical role in cell elongation (Vidali et al. 2010). In contrast, single myosin XI mutants (*myosin XI-I*) in Arabidopsis have more subtle phenotypes: nuclear migration defects that generate aberrant division planes during stomatal development that reduce stomatal density (Muroyama et al. 2020) and nuclear shape and movement defects (Tamura et al. 2013).

Defects in nuclear migration/polarization during subsidiary cell development were not detected in the *o1* mutant in maize, but both ER organization and subsidiary cell division positioning were aberrant (Wang et al. 2012; Nan et al. 2023). The *o1* mutant was originally identified due to the opaque kernel phenotype, which is caused by aberrant protein body accumulation in the endosperm (Wang et al. 2012). The O1 protein associates with the ER and also as puncta in the cytoplasm and in the phragmoplast midline (Wang et al. 2012; Nan et al. 2023). Overexpression of the tail domain disrupts ER motility in tobacco cells (Wang et al. 2012).

Plant class XI myosins are processive motors that dimerize and transport cargo and promote cytoplasmic streaming (Tominaga et al. 2003; Tominaga and Nakano 2012). Overexpression or ectopic expression of truncated myosins lacking the motor domain has a dominant-negative effect that impairs myosin activity in vivo, potentially due to dimerization (Avisar et al. 2008, 2009; Peremyslov et al. 2008; Sparkes et al. 2008; Wang et al. 2012; Stephan et al. 2021). As a motor, Arabidopsis myosin XI-I has unique features, including a high affinity for actin with corresponding low velocity and ATPase activity compared to other myosin XIs (Haraguchi et al. 2016). Its closest homolog in maize is O1, and although O1's motor activity has not been characterized, it is plausible to speculate that homodimers and heterodimers including functional O1 may possess a similarly limited motor activity. Overall, individual myosins are likely to have different actin binding affinities, cargo, and velocities, and therefore different (but potentially overlapping) cellular functions. These diverse cellular functions may contribute to the control of different phenotypes at the tissue, organ, and plant levels. Previous immunoprecipitation experiments show that O1 interacts with other myosins, including myosin XI-K, XI-F, and XI-G family members as well as multiple myosin VIIIs (Nan et al. 2023). Myosin VIII (ATM1) from Arabidopsis has 10-fold lower motility than many myosin XIs and may instead play roles in tethering or tension sensing (Haraguchi et al. 2014). Myosin VIII and myosin XI-1 from *Selaginella moellendorffii* showed similar slow velocities compared to ATM1 when expressed heterologously in *Nicotiana benthamiana* (Tachibana et al. 2026); thus, O1-myosin VIII heterodimers may be slow moving or nonmotile. Notably, *O1* has now been identified in 4 genetic screens, with 3 distinct phenotypes (Wang et al. 2012; Nan et al. 2023). To the best of our knowledge, no other maize gene encoding a myosin has been identified in a forward genetic screen. This implies that O1's function may be unique—either because of its intrinsic properties, such as binding affinity or cargo—or because it heterodimerizes with many different myosins. O1 also interacts with actin binding proteins, heat shock interacting proteins, and kinesins that are related to PHRAGMOPLAST ORIENTING KINESIN1 (POK1) and POK2 (Müller et al. 2006; Wang et al. 2012; Nan et al. 2023). Determining the scope of motility, dimerization, and cargos across different myosins in future studies may help elucidate O1 and myosin function in general.

**Figure 7:**
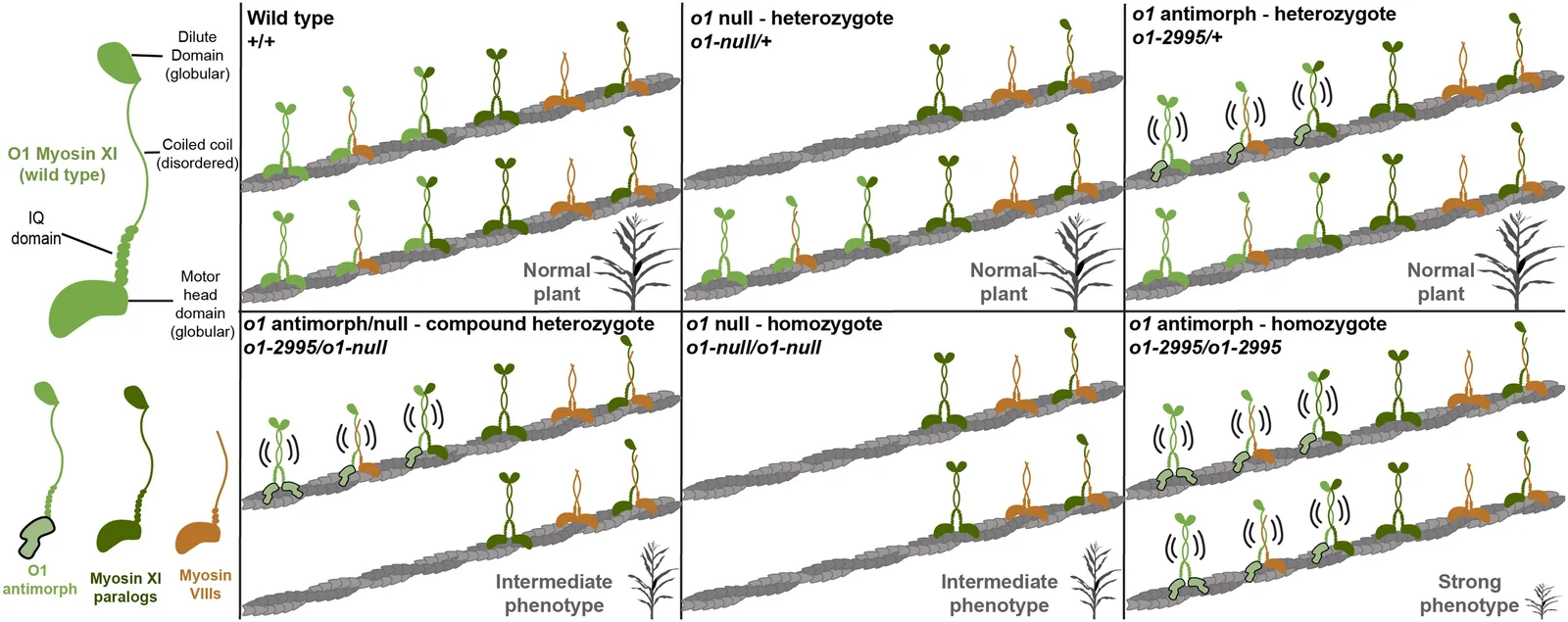
Model of O1 cellular function across different maize genotypes. Myosin XI (green) and VIII (brown) polypeptide monomers contain a motor head, a neck region containing IQ domains (with more IQ domains in myosins XI than VIII), and a tail region with coiled-coil domains (less extensive in myosins VIII) involved in cargo binding. Myosin XI monomers (including XI-I, ie O1, light green) also contain a dilute domain in the tail region. The defective motor head of the O1 antimorph monomer is indicated as misshapen with a solid outline. Each main panel illustrates a genotype and its relevant, expressed myosin dimers bound to actin microfilaments (gray). Naked dimers (without annotation) would be fully functional. The double parentheses annotation depicts antimorph-containing dimers with hypothesized, disturbed activity, eg with compromised motility, tethering, tension sensing, and/or cargo binding, as discussed in the text. Similarly, antimorph-containing dimers may or may not bind to actin. Top left) In WT plants, O1 homodimerizes and heterodimerizes with other myosin XI isoforms and with myosin VIII. Relative numbers of heterodimers and homodimers in vivo are unknown; this panel illustrates 2 “copies” of each dimer type. Each myosin dimer will have associated cargo (not shown), which could be specific or shared with other dimers. Top center) In *o1* null heterozygotes, the frequency of dimers containing O1 decreases, but all dimer types are present. The plants are phenotypically WT. Bottom center) Complete loss of O1 in *o1* null mutants eliminates any complex containing O1, leading to fewer dimer types and an intermediate plant phenotype. Top right) In *o1* antimorph heterozygotes, O1-2995 or O1-tan62 proteins have defective head domains, leading to “poisoned” complexes (with double parentheses). Nonetheless, these alleles behave as fully recessive and not as dominant negatives, ie heterozygotes are phenotypically normal, plausibly because some nonpoisoned homodimers and heterodimers form which are sufficient for function. O1-2995 homodimers are also present but not shown. Bottom right) In antimorph homozygotes, there are only disturbed-function O1 homodimers and more all O1-containing heterodimers are poisoned, leading to a stronger phenotype than the null mutants. This may be due in part to disabling of functional complexes (ie binding to other myosins) or to sequestering cargo (ie binding to other proteins, not shown). Bottom left) Plants that have 1 antimorph allele and 1 null allele have an intermediate phenotype like the null homozygote, suggesting a dosage requirement for poisoned complexes to manifest a strong phenotype.

Our current hypothesis is that both missense mutant alleles described in this paper generate proteins with impaired function of a motor domain activity, such as actin binding or the ATPase activity required for movement. We predict that O1-2995 and O1-TAN62 mutant proteins, despite predicted defects in motor domain function, would still be capable of interacting with multiple other proteins, including both myosin XIs and myosin VIIIs (Nan et al. 2023), and cargoes, as outlined in our speculative model (Fig. 7). Given the array of possible dimers involving O1, the model is relevant to all of those potential dimer functions including, eg motility, tethering, tension sensing, and cargo binding. The *o1-2995* mutant is recessive: no antimorph effect is seen in either simple (*O1/o1-2995*) or compound (*o1-ref/o1-2995*) heterozygous plants. We speculate that the recessive nature of *o1-2995* reflects insufficient expression levels of the mutant form of the protein. Thus, the more severe mutant phenotype of homozygous *o1-2995* or *o1-tan-62* relative to *o1* null mutants could be due to O1 mutant proteins binding to and reducing activity of other myosins and/or to the dynamics of competition for binding partners in general, including cargo (Fig. 7). It is plausible that the antimorph effects are observed only in homozygous o1-2295 genotypes due to dosage effects, ie increased expression of *o1-2995* coupled with the absence of functional O1 may sufficiently increase the relative abundance of poisoned, O1-containing protein complexes to enhance the plant growth defects only minimally evident in *o1* null mutants.

Severity of the subsidiary cell division defects correlated with defects in plant height, but the relationship between cell division defects and plant height is unclear. One potential hypothesis is that gas exchange defects might lead to short plants. However, while aberrant subsidiary cells disrupt the closure of guard cells, either mild or no gas exchange defects were observed in other mutants with aberrant subsidiary cells including *pangloss1* (*pan1*), *pan2*, or the *pan1 pan2* double mutant (Liu et al. 2024). Alternative and more plausible hypotheses posit that O1 has multiple functions: one required for cell expansion and another that affects subsidiary cell division. Cell expansion defects could be enhanced by additional mechanical stresses generated via aberrantly shaped cells caused by division positioning defects (Sampathkumar et al. 2014).

## Materials and methods

### Genetic stocks and phenotypic characterization

The rsl*-12.2995 (*o1-2995*) mutant allele originated from a *ramosa1* (*ra1*) modifier screening population generated via EMS pollen mutagenesis. *ra1-63* was backcrossed 6 times to Mo17 to create a homozygous mutant stock. Pollen from mutants was collected, treated with 0.06% EMS in paraffin oil as described (Weeks 2013), and used to pollinate the same genotype. We screened for dominant modifiers in the M1 generation, and then the remaining plants were self-fertilized. The resulting M2 populations were screened for recessive modifiers, where we identified rsl*-12.2995 (*o1-2995*) as a mutant that segregated 3:1 and displayed short plant stature with suppressed inflorescence branching.

To understand its penetrance and expressivity in diverse genetic backgrounds, we backcrossed the *o1-2995* allele to B73, Mo17, and W22 inbred lines. B73-introgressed stocks for other mutants were sourced from the authors’ labs for *ra1-R* (EV), *ra2-R* (EV), *o1-ref* (MF), and *o1-N1242A* (MF) or from David Jackson (Cold Spring Harbor Laboratory) for *ra3-ref*. Alleles of *o1-ref* and *o1-N1242A* were obtained from the Maize Genetics Cooperation Stock Center and introgressed into B73 4 times before crossing to *o1-2995*.

*o1-tangled62* (*o1-tan62*) was identified from an EMS mutagenesis performed on B73 kernels. The M0 generation was open-pollinated, followed by self-fertilization of the M1 generation. Glue impressions of leaf 2 or 3 in the M2 generation were examined for division plane positioning defects. *o1-tan62* was backcrossed to B73 at least 3 times before sequencing the *O1* locus.

### Map-based cloning, sequencing, linkage analysis, and complementation test

To identify the causative mutation lesion responsible for the rsl*-12.2995 (*o1-2995*) mutant phenotype (Zebosi 2022), we used map-based cloning methods with an F2 mapping population developed between Mo17 and B73.

A BSA-GBS method was devised and implemented as follows: a [B73 × Mo17] F2 mapping population was sown, and leaf tissue samples were collected as 2 pools bulked from mutant (*n* = 12) and normal sibling (*n* = 26) plants. 150 to 300 leaf punches (6 mm) were collected for each pool, with an equal number of punches collected from each individual plant in the bulked sample. High molecular weight genomic DNA was extracted using a urea-based protocol (Chen and Dellaporta 1994). Genotyping-by-sequencing (Elshire et al. 2011) was then adapted and applied to the DNA extracted from the 2 bulked pools (see Kokulapalan (2018) for more detail). Briefly, genomic DNAs were quantified using the Promega Quantifluor dsDNA system and used in the BSA-GBS bench protocol consisting of 3 main steps, namely, restriction digestion using ApeKI, ligation of a barcoded and a common adapter, and PCR amplification and cleanup to construct the barcoded sequencing library. Sequencing was performed using 100-bp single-end sequencing with Illumina HiSeq 2500 (Iowa State University [ISU], DNA facility). Sequencing reads were demultiplexed and separated into independent files for each bulked pool and clipped, all using fastq-mcf (Aronesty 2013). Reads were aligned to maize B73 RefGen_AGPv4 using minimap2 (Jiao et al. 2017; Li 2018). Euclidean distance (ED) between the normal and mutant pools was calculated using nucleotide composition and used to calculate ED4 to suppress noise. ED4 distances were plotted on a Manhattan plot using ggplot2 to visualize differences between the pools (Wickham 2016). BSA-GBS roughly mapped the *o1-2995* mutation to the long arm of chromosome 4 (Fig. S2), within a peak containing ∼989 genes.

Using publicly available molecular markers, we further mapped the mutation to a region between markers IDP2 (18 recombinants, 2.24 cm) and IDP8018 (41 recombinants, 5.11 cm), and then with public SNPs to a region between BZ2995_2 (8 recombinants, 1 cm) and BZ2995_1 (2 recombinants, 0.25 cm), comprising a physical distance of 1.5 Mb and 28 genes (Fig. 2a). To further narrow down the location of the causal mutation, we used 2 fine-mapping populations (F2 and F1BC1) constructed by crossing *o1-2995* mutants in the original Mo17 genetic background to the B73 inbred line. DNA was extracted as previously described (Zebosi et al. 2025). Using publicly available insertion/deletion polymorphisms between B73 and Mo17, we designed new markers to reduce the fine-mapping to the interval between IDP2 (18/802, 2.24 cm) and IDP8018 (41/802, 5.11 cm). To further narrow the interval, SNPs were used to design new markers that localized the mutation to a 1.5 Mb interval between BZ2995_6 (8/802, 1 cm) and BZ2995_1 (2/802, 0.25 cm) with 28 genes (Fig. 2a). In nonrepetitive regions between markers BZ2995_6 and BZ2995_1, B73 and Mo17 contained no DNA sequence polymorphisms to advance the fine-mapping process. We therefore performed WGS of a single mutant plant to identify EMS-induced SNPs in the interval for marker development and identifying potential candidate genes. The library was prepared and sequenced on an Illumina HiSeq by Novogene Inc., UC Davis, California, to generate 50 Gb of 150-bp paired-end sequencing data. The raw reads were subjected to quality control using FASTQC (Andrews 2010) and adapters, and reads with low base quality were trimmed using Trimmomatic version 0.32 (Bolger et al. 2014). The trimmed reads were aligned to the Mo17 genome using the “mem” algorithm in BWA-MEM version 0.7.17 (Li and Durbin 2010), and the resulting sequence alignment SAM files were converted to BAM files and filtered for uniquely mapped reads using Samtools (Li et al. 2009). SNP calling against the Mo17 genome was performed using the Samtools mpileup command, and homozygous non-Mo17 or EMS SNPs (G > A and C > T) in the 1.5 Mb interval were identified. We identified 3 genes with exonic EMS SNPs, GRMZM2G521481, GRMZM2G091143, and GRMZM2G449909 (*Opaque1/O1*).

From the EMS-like SNPs, we developed new markers and used them in F2 populations consisting of 2,289 plants (both WT and mutants) to find 6 recombination events between the 3 candidate genes. Left-side recombinants (3 and 17) and right-side recombinants (9 and 16) suggested that the causal mutation was within a physical distance of ∼0.6 Mb that contained only one gene, the previously cloned *Opaque1* (*O1*) (Fig. 2b). We therefore phenotyped kernels from our segregating populations and found an opaque kernel phenotype cosegregated with the *o1-2995* mutants. We verified the 6 recombinants by test crossing each with *o1-2995* mutants and phenotyping the progeny for kernel opaqueness and plant height. To validate *Opaque1* as the causal locus, we performed a complementation test by crossing *o1-2995* mutant to 2 *o1* mutant alleles (*o1-ref/+* and *o1-N1243/+*).

To sequence the *O1* coding sequence in *tan62* mutants, RNA was extracted from young leaf tissue of *o1-tan62* homozygous mutants and WT plants using the RNeasy Plant Mini Kit (QIAGEN) and reverse transcribed into cDNA using the QuantiTect Reverse Transcription Kit (QIAGEN). Then, the cDNA was amplified into 2 halves and sequenced using primers in Table S1. PCR products were run on a gel and bands were gel purified using the QIAquick Gel Extraction Kit (QIAGEN). Seven PCR products spanning the entire coding sequence were sent for Sanger sequencing at the Genomics Core Facility (University of California, Riverside). *o1-tan62* and WT sequences were compared to each other and GRMZM2G449909 (*O1*) by using A plasmid Editor (Davis and Jorgensen 2022) and NCBI BLAST (Altschul et al. 1990). DNA from an additional 3 *tan62* mutants was extracted, and sequenced PCR products confirmed the mutation. Complementation test crosses between *o1-tan62* and *o1-N1242A* homozygous mutants were done in the field at the University of California, Riverside in the Summer of 2021. Approximately 50 kernels each from 11 independent crosses were assessed for and had opaque kernel phenotype confirming that *tan62* was allelic to *o1*.

### Plant growth conditions

Field-grown plants were grown in the summer maize nursery at Curtiss Farms, ISU in Ames, Iowa or at University of California, Riverside (UCR) Agricultural Operations. UCR greenhouse growing conditions: maize kernels were planted in 3.8 L pots with soil (20% peat, 50% bark, 10% perlite, and 20% medium vermiculite) supplemented with magnesium nitrate (50 ppm N and 45 ppm Mg), calcium nitrate (75 ppm N and 90 ppm Ca), and Osmocote Classic 3 to 4 M (NPK 14-14-14%, AICL SKU #E90550) with standard greenhouse conditions (31 to 33 °C daytime temperature with supplemental lighting from 17:00 to 21:00 PM at ∼ 400 µE m^−2^ s^−1^) (Uyehara et al. 2024).

### Phylogenetic and gene expression analysis

Myosin amino acid sequences of Arabidopsis, maize, rice, Setaria, sorghum, and Physcomitrella were obtained from Phytozome (Goodstein et al. 2012). Sequence alignments were performed using ClustalW2 (Larkin et al. 2007) and Mesquite software (Maddison and Maddison 2023). Approximate maximum-likelihood phylogenetic trees were constructed as described by Zebosi et al. (2024). Raw RNA sequence reads from several tissues were downloaded from the short read archive (https://www.ncbi.nlm.nih.gov/sra), and reads were analyzed as described (Zebosi et al. 2024).

### Protein analysis

Membrane proteins were extracted from the basal 0.5 to 3 cm of leaves from the 4-leaf stage and separated by SDS-PAGE, according to Facette et al. (2015). The previously described rabbit antibody was generated by injection of O1-specific peptides (Nan et al. 2023) and used at 0.66 *μ*g/mL. Across the last 3, well-annotated genome assemblies of maize B73 (RefGen_v3, v4 and v5), up to 23 splice variants are predicted for the *O1* gene. Among them, Zm00001d052110_T002 (b73refgenv4) produces a protein of 1,013 amino acids (116 kDa), similar to the size identified in our immunoblot. Previously published co-immunoprecipitation with mass spectrometry (co-IP/MS) and immunoblot data with this OPAQUE1 antibody detected at least 2 splice isoforms in developing leaves.

### Phenotypic characterization

Phenotypic characterization was done using the *o1-2995* allele or the *o1-tan62* allele introgressed in B73 compared to their corresponding WT siblings. Agronomic traits, such as plant height, total leaf number, leaf length and width, tassel length, tassel, and EBN, were characterized using segregating populations. Plant height measurements were taken by measuring from the soil surface to the tip of the tassel at maturity. The total leaf number was tracked from germination and measured in mature plants. The peduncle length was measured from the flag leaf node to the lowest tassel branch. The tassel length was measured from the lowest tassel branch to the tip of the tassel. The internode length and diameter and peduncle lengths were measured with a tape measure. For internode length, measurements were taken starting from the first internode closest to the tassel proceeding basipetally to the 12th internode from the tassel.

### Phloroglucinol–HCl staining

For phloroglucinol–HCl staining, midribs from adult leaf 10 were hand-sectioned with a double-edge razor, and sections were stained using a phloroglucinol–HCl staining solution (Strable et al. 2017). The stained sections were observed under a dissecting microscope (Leica MZ125) and imaged using a digital camera.

### Leaf epidermal impressions

Leaf epidermal impressions of the abaxial surface were produced from mid-length between the ligule and leaf tip and mid-way between the leaf margin and the mid-vein using cyanoacrylate glue as described (Allsman et al. 2019). The slides with the epidermal imprints were observed under the Olympus BX60 light microscope and imaged using a digital camera (Jenoptik C5). The size of epidermal cells and the number of aberrant subsidiary cells were quantified from 3 random viewable fields using ImageJ (Rueden et al. 2017). For *o1-tan62* mutants and WT siblings, leaf epidermal impressions were taken from the abaxial side of the second leaf. Epidermal imprints were imaged with a light compound microscope (Nikon) with digital microscope camera attachment (AmScope MD130).

### Confocal microscopy

Images were taken of the asymmetric divisions of the subsidiary mother cell using a Yokogawa W1 spinning disk on a Nikon Eclipse TE inverted stand microscope with an EM-CCD camera (Hamamatsu 9100c). The ASI Peizo stage and 3 axis DC servo motor controllers were controlled using Micromanager software (www.micromanager.org). Solid-state Obis lasers (power from 40 to 100 mW) were used in combination with standard emission filters (Chroma Technology). For CFP-β-TUBULIN, a 445 laser with emission filter of 480/40 was used. For YFP-ɑ-TUBULIN, a 514 laser with emission filter of 540/30 was used. *o1-2995*, *o1-tan62*, and their WT siblings, expressing CFP-β-TUBULIN or YFP-ɑ-TUBULIN (Mohanty et al. 2009), were grown in the greenhouse under standard conditions as described (Uyehara et al. 2024). Four week-old plants were dissected to an emerging leaf with ligule height <2 mm, and the asymmetric dividing zone (within 1.5 cm from the ligule) was mounted in a rose chamber (Rasmussen 2016). Micrographs of asymmetric divisions of the subsidiary mother cell were captured along the entirety of the section and subsequently analyzed using the Fiji version of ImageJ (Schindelin et al. 2012).

### Data analysis and figure preparation

Graphs and statistics were done using the R software environment for statistical computing and graphics (https://www.R-project.org/) and RStudio software (https://posit.co/) using several packages. Some figures were assembled using the Gnu Image Manipulation Program (Gimp, version 2.10.38, https://www.gimp.org).

### Accession numbers

*Opaque1 (O1):* GRMZM2G449909 (B73 RefGen_v3), Zm00001d052110 (Zm-B73-REFERENCE-GRAMENE-4.0), Zm00001eb193160 (Zm-B73-REFERENCE-NAM-5.0). *Ramosa1 (Ra1):* GRMZM2G361210 (B73 RefGen_v3), Zm00001d034642 (Zm-B73-REFERENCE-GRAMENE-4.0), Zm00001eb062570 (Zm-B73-REFERENCE-NAM-5.0). *Ramosa2 (Ra2):* AC233943.1_FG002 (B73 RefGen_v3), Zm00001d039694 (Zm-B73-REFERENCE-GRAMENE-4.0), Zm00001eb123060 (Zm-B73-REFERENCE-NAM-5.0). *Ramosa3 (Ra3):* GRMZM2G014729 (B73 RefGen_v3), Zm00001d022193 (Zm-B73-REFERENCE-GRAMENE-4.0), Zm00001eb327910 (Zm-B73-REFERENCE-NAM-5.0).

## Supplementary Material

kiag605_Supplementary_Data

## Data Availability

All data supporting the findings of this study are provided within the article and its supporting information.
