# Supplementary material for: Recessive antimorph alleles reveal novel functions of the Opaque1 myosin XI in maize
Source: Plant Physiol. 2026 Aug 20;202(1):kiag605. doi: 10.1093/plphys/kiag605 (PMC13579333; doi:10.1093/plphys/kiag605)
Supplement: Supplementary file 1 [file kiag605_supplementary_data.zip › 2995 paper supp table 07312026.pdf]

Inbred	Genotype	n	Height	SD	SE	CI	CV %
Mo17-8	WT	47	232.8	13.0	1.9	3.8	5.6
Mo17-8	*o1-2995*	39	192.0	11.5	1.8	3.7	6.0
B73-6	WT	20	229.2	12.5	2.8	5.9	5.5
B73-6	*o1-2995*	17	114.1	14.7	3.6	7.6	12.9
W22-2	WT	31	229.4	16.3	2.9	6.0	7.1
W22-2	*o1-2995*	39	150.0	18.7	3.0	6.0	12.4

Supplementary Table 1. Plant height measurement data for normal siblings (WT) and *o1-2995* mutants represented in Figure 1B. In the Inbred column the hyphenated number indicates the number of backcrosses e.g. Mo17-8 implies the line was backcrossed eight times to Mo17 before phenotyping. Other data include sample size (n), mean height, standard deviation (SD), standard error (SE), 95% confidence interval (CI) and coefficient of variation (CV).
